# Cannabinoid Hyperemesis Syndrome: A Paradoxical Cannabis Effect

**DOI:** 10.1155/2015/405238

**Published:** 2015-07-22

**Authors:** Ivonne Marie Figueroa-Rivera, Rodolfo Estremera-Marcial, Marielly Sierra-Mercado, José Gutiérrez-Núñez, Doris H. Toro

**Affiliations:** ^1^Internal Medicine Program, Department of Internal Medicine, Veterans Affairs Caribbean Healthcare System, San Juan, PR 00921-3201, USA; ^2^Department of Gastroenterology, Veterans Affairs Caribbean Healthcare System, San Juan, PR 00921-3201, USA

## Abstract

Despite well-established antiemetic properties of marijuana, there has been increasing evidence of a paradoxical effect in the gastrointestinal tract and central nervous system, given rise to a new and underrecognized clinical entity called the Cannabinoid Hyperemesis Syndrome. Reported cases in the medical literature have established a series of patients exhibiting a classical triad of symptoms: cyclic vomiting, chronic marijuana use, and compulsive bathing. We present a case of a 29-year-old man whose clinical presentation strongly correlates with cannabinoid hyperemesis syndrome. Despite a diagnosis of exclusion, this syndrome should be considered plausible in the setting of a patient with recurrent intractable vomiting and a strong history of cannabis use as presented in this case.

## 1. Introduction

Cannabis—commonly known as marijuana—is the recreational drug most widely used in the United States and worldwide [1]. It has been used in the medical profession as an antiemetic and an appetite stimulant; however, heavy prolonged use has been shown to cause the poorly understood paradoxical Cannabinoid Hyperemesis Syndrome (CHS). Cannabinoid Hyperemesis Syndrome was first described in 2004; since then, at least 31 cases have been reported in the medical literature [2]. Clinical presentation is characterized by severe nausea and vomiting, colicky abdominal pain, long-term cannabis use (generally over 5 years), and compulsive hot bathing [1–4]. The later has been described in up to 98% of the cases and has been considered pathognomonic [5]. Symptoms of CHS resolve upon abstaining from cannabis use.

## 2. Case Report

A 29-year-old male patient with medical history of anxiety disorder, major depression, asthma, and substance abuse presented to the emergency department with a 3-day history of nausea and vomiting. He reported having intermittent episodes of intractable vomiting for the past year that has required multiple visits to the emergency department and hospital admissions. These episodes were associated with diffuse colicky abdominal discomfort, repetitive episodes of vomiting of gastric content, decreased oral intake, and a 30-pound weight loss during the past three months. He denied fever, chills, recent travels, sick contacts, changes in bowel habits, hematemesis, melena, or hematochezia. There were no exacerbating factors or triggers, however, symptoms were temporarily relieved with frequent hot showers.

Physical exam revealed a thin young man. His abdomen was soft and depressible, nondistended, diffusely tender without guarding, rebound, or rigidity. Laboratories upon admission showed no leukocytosis, normal hemoglobin levels, and platelet count. Chemistry panel showed neither electrolytes nor acid-base disturbances. Liver and pancreatic enzymes were found within normal limits. He denied alcohol and tobacco use but, reported active use of marijuana for the past 16 years, smoking at least three to four joints a day. Toxicology screening was positive for cannabis at the time of admission.

The patient was admitted to internal medicine ward and gastroenterology service was consulted for further recommendations. In view of the patient's cyclic vomiting pattern, repetitive admissions, and significant weight loss, extensive workup was conducted to exclude other causes of these alarming signs and symptoms. An abdominopelvic CT scan and a brain MRI were essentially normal. Upper endoscopy revealed only mild gastritis without evidence of* Helicobacter pylori* infection, while colonoscopy was normal. A gastric emptying study was performed with 99mTc-Sulfur Colloid labeled meal. Results demonstrated a markedly diminished and delayed emptying of the stomach's contents into the small bowel with a calculated gastric retention of 100% at 1 hour. At 3 and 4 hours, gastric retention was 57% and 45%, respectively. Calculated gastric emptying half-time was 188 minutes, results that were compatible with gastroparesis.

Antiemetic therapy and IV hydration did not relieve his symptoms. During hospitalization he was repeatedly found taking hot showers which he reported to provide transient relief. Psychiatry consultant recommended intravenous lorazepam resulting in significant improvement. Within the first 72 hours of admission his compulsive hot bathing as well as the gastrointestinal symptoms began to abate. After a 4-day hospital course, the cessation of cannabis use, and eventual tolerance of oral intake, the patient was successfully discharged home.

## 3. Discussion

Cannabinoid Hyperemesis Syndrome is a clinical condition first described in 2004 [6]. In 2009, after several case reports and published articles, a set of clinical characteristics was proposed for diagnosing this syndrome [7]. These include the following: long standing use of cannabis, severe cyclic nausea and vomiting, colicky abdominal pain, and relief of symptoms with hot baths and cannabis use cessation, in the absence of gallbladder or pancreatic inflammation.

This syndrome is a recurrent disorder in which three phases have been described: a prodromal, hyperemetic, and a recovery phase [1, 5]. The pathogenesis of paradoxical hyperemesis symptoms remains to be an enigma and potential mechanisms associated with cumulative and toxic effects of Δ_9_-tetrahydrocannabinol (Δ_9_-THC) have been suggested [5]. Δ_9_-THC is the principal active compound in cannabis. The isolation of this major psychoactive ingredient led to the understanding of the site of action of cannabis and ultimately to the identification of CB_1_ and CB_2_ receptors in the central and enteric nervous system [8]. Through animal models, cannabinoids spectrum of activity has been identified and it is known to be an agonist of CB_1_ receptors. Through stimulation of these presynaptic receptors, the release of emetic neurotransmitters is inhibited, thus preventing vomiting [9]. Based on the known antiemetic effect of Δ_9_-THC, the underlying mechanism of the hyperemesis in CHS is not well understood. Pharmacokinetic and pharmacodynamic factors associated with the chronic exposure to Δ_9_-THC have been postulated as being responsible for the hyperemesis [9]. Some components of cannabis have a long half-life which may accumulate in the brain causing buildup of toxic chemicals. At the same time, it is speculated that this buildup downregulates cannabinoid receptors due to a high exposure to the ligand. In the presence of reduced functional CB_1_ receptors, the agonist nature of Δ_9_-THC can transform into antagonist action [8]. CB_1_ receptors exert a neuromodulatory role in the gut and cannabinoids stimulation is known to slow gastric emptying and peristalsis. In patients with CHS, this activity is thought to override the brain CB_1_ antiemetic effect [3, 5]. CB_1_ receptors are also found near the thermoregulatory center of the hypothalamus which could explain the compulsive hot bathing [3, 5]. This has been thought to be related to counteraction of cannabis-induced disequilibrium at the thermoregulatory center [3, 5]. In addition, hot bathing may contribute to CHS symptom relief by inducing splanchnic vasodilation which causes redistribution of the blood from the gut to the skin causing a “cutaneous steal syndrome” [5].

To our knowledge, this is the second case report of cannabinoid hyperemesis in the Caribbean and the first case reported in Puerto Rico [10]. Few case reports have been described in the medical literature. In a large retrospective study, 6% of a cohort of 1571 patients admitted with unexplained recurrent vomiting and reported cannabis use met the inclusion criteria for CHS, suggesting that this entity is mostly underrecognized and underdiagnosed [11].

Prompt recognition can reduce costs associated with unnecessary workups, ER visits, and hospital admissions. Future studies are needed to better understand the etiology, prevalence, and risk factors for developing this syndrome.
